# Comparison of Effects of Ultrasound Therapy and Nerve-Gliding Techniques on Patients with Carpal Tunnel Syndrome: A Randomized Clinical Trial

**DOI:** 10.5812/aapm-147159

**Published:** 2024-07-07

**Authors:** Maryam Nazarian, Maryam Sadat Rahimi, Alireza Ghanbari, Seyed Alireza Ghoreishi

**Affiliations:** 1 Student Research Committee, School of Medicine, Birjand University of Medical Sciences, Birjand, Iran; 2 Physical Medicine and Rehabilitation Specialist, Cardiovascular Diseases Research Center, Birjand University of Medical Sciences, Birjand, Iran; 3 Department of Orthopedic Surgery, School of Medicine, Iran University of Medical Sciences, Tehran, Iran; 4 Orthopedic Department, Imam Reza Hospital, Mashhad University of Medical Sciences, Mashhad, Iran

**Keywords:** Ultrasound, Nerve-Gliding Techniques, Carpal Tunnel Syndrome, Treatment

## Abstract

**Background:**

Carpal tunnel syndrome (CTS) is a common neuropathy caused by pressure on the median nerve in the wrist, affecting 1% to 5% of the population. Treatment options include pharmacologic management, rest, splints, local steroid injections, and physical therapy. Conservative treatments, such as ultrasound therapy and nerve-gliding exercises, can also be effective.

**Objectives:**

This study aimed to compare the effects of ultrasound therapy and nerve-gliding exercises on reducing symptoms, improving hand function, and electrodiagnostic tests in patients with CTS.

**Methods:**

This randomized clinical trial was conducted on patients with CTS at the specialized physical medicine and rehabilitation clinic at Birjand University of Medical Sciences. The study included 48 patients with CTS, divided into two groups. The ultrasound group received therapy at a frequency of 1 MHz and a current intensity of 1 watt/cm², with each session lasting 5 minutes. The nerve-gliding group underwent 10 sessions of treatment, three times a week. Pain intensity was evaluated using VAS criteria, symptom intensity using the BOSTON Questionnaire, and median nerve latency with EMG-NCS.

**Results:**

The study included 48 patients with CTS, divided into two groups. Both groups showed improved severity of symptoms and function scores at the end of the study (P < 0.001). The ultrasound group significantly reduced the sensory and motor median nerve latency scores (P < 0.001 and P = 0.001, respectively), and the pain score diminished significantly in both groups (P < 0.001).

**Conclusions:**

Ultrasound and neural-gliding techniques are effective in reducing patients' symptoms and pain intensity in the short term. Additionally, ultrasound can improve electrodiagnostic indicators.

## 1. Background

Carpal tunnel syndrome (CTS) is a common neuropathy caused by pressure on the median nerve in the wrist (1). The carpal tunnel is a rigid opening in the forearm that connects the forearm to the palm (2). The tunnel is formed by the carpal bones, where the normal pressure is 2.5 mmHg. In CTS, the pressure can increase up to 32 mmHg, leading to symptoms such as pain and numbness in the thumb, index, middle, and outer half of the ring finger, especially at night (3-6). Carpal tunnel syndrome affects 1% to 5% of the population, with women aged 40 - 60 being 3 to 10 times more likely to develop it. Manual workers are also at higher risk (7-9). The most common cause of CTS is unknown, but activities involving excessive wrist use, such as bending and straightening the hand, are associated with its development (10, 11). Other factors that increase the risk of CTS include diabetes, obesity, hypothyroidism, pregnancy, and certain diseases such as rheumatoid arthritis (12).

Carpal tunnel syndrome can lead to permanent sensory and motor disorders if left untreated. Diagnosis is based on physical examination and electrodiagnostic tests (13). Continuous increased pressure in the carpal tunnel, along with direct damage to the nerves and vessels of the perineurium, causes a decline in the conduction speed of the nerve, ischemia, weakness, and atrophy of the thenar muscles (14). Failure to treat the disease promptly can lead to permanent sensory and motor disorders, reduced power and quality of work, increased treatment costs, and early disability (15). Patients with this syndrome experience the highest degree of disability in fine handwork (16).

Treatment aims to control pain, improve neuromuscular function, and reduce disease progression. Conservative treatment options for mild to moderate CTS include medication therapy, rest, use of splints, local steroid injections inside the carpal tunnel, and physical therapy methods such as ultrasound management, nerve gliding exercises, and massage (17, 18). Ultrasound management is a safe method that uses sound waves to relieve pain and improve blood flow. Nerve gliding exercises involve moving the median nerve to reduce adhesion and improve oxygenation, which have been found to be effective in treating CTS (19-21).

Despite the numerous treatments available for CTS, there is still no consensus on the best treatment. Some studies have shown that ultrasound and nerve gliding techniques are effective in reducing the symptoms of CTS, but conflicting results have also been reported (22, 23).

## 2. Objectives

Therefore, further studies are needed to determine the effectiveness of these treatments. This study aimed to compare the effects of ultrasound management and nerve-gliding exercises on reducing the symptoms in CTS patients.

## 3. Methods

**Figure 1.**
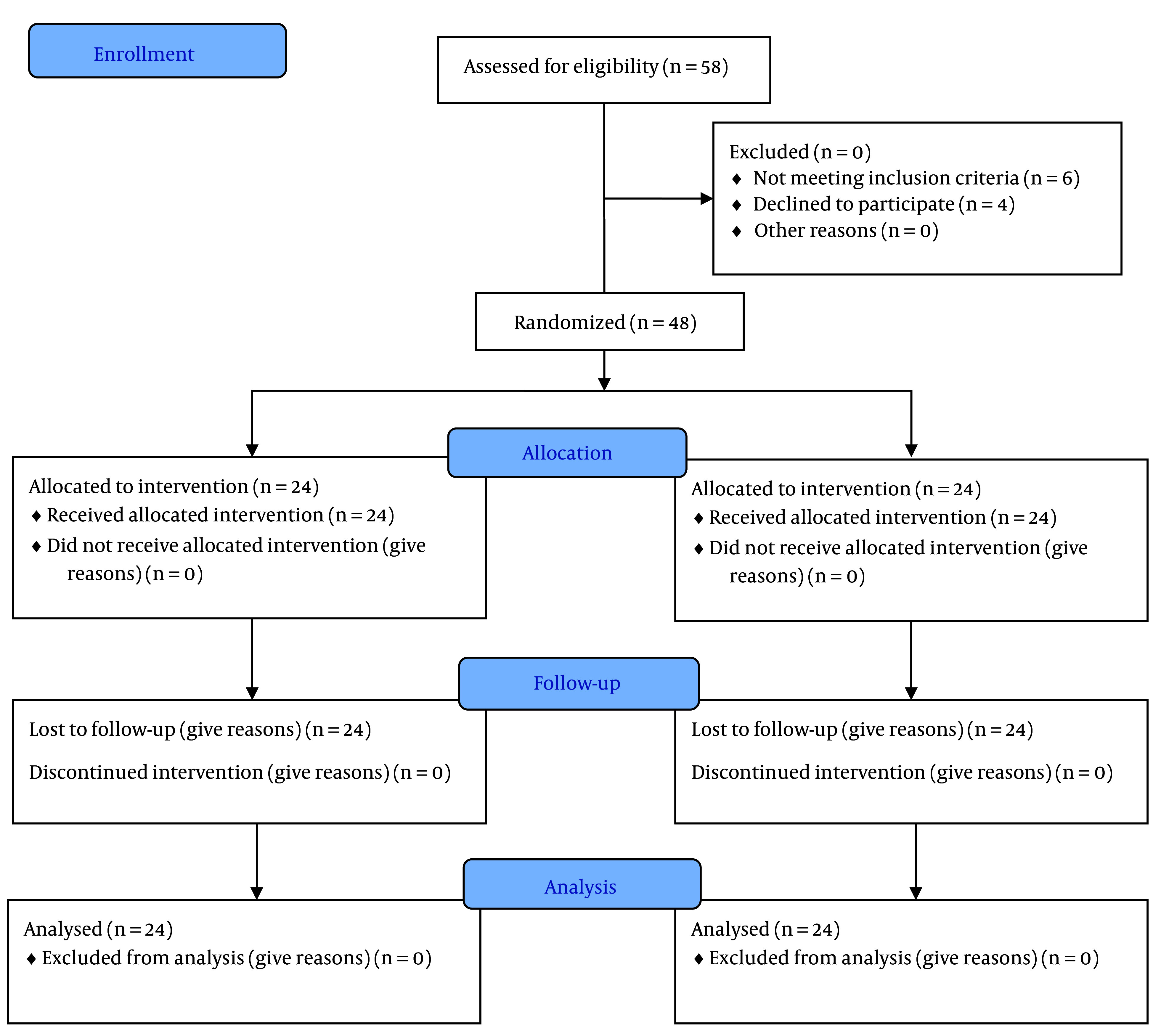
CONSORT diagram

This randomized clinical trial was conducted on patients with CTS who were referred to the specialized physical medicine and rehabilitation clinic at Birjand University of Medical Sciences. This study was registered in the Iranian Registry of Clinical Trials (IRCT) (clinical trial registration code: IRCT20190618043934N23). The inclusion criteria were patients suffering from CTS with a mild to moderate form of the disease based on electrodiagnostic criteria, no use of non-steroidal anti-inflammatory drugs (NSAIDs) or steroids during the intervention, and a stable mental state to participate in the study. The exclusion criteria included pregnancy, previous treatment with ultrasound or laser, regular use of painkillers and anti-inflammatory medications, use of local steroid injections inside the carpal tunnel within the last year or during the study, severe atrophy of the thenar region, a history of malignant tumors, peripheral system neuropathies, and irregular attendance during the treatment process (Figure 1). The sample size was calculated using the study conducted by Atya and Mansour (24) and the following formula. Considering the time-consuming process of treatment, the need for patient follow-up, prevention of heterogeneity in the study groups, and accounting for a 20% dropout rate, the sample size in each group was estimated to be 24 hands/patients.


$$n=\frac{\;{\left(Z_{1-\frac{\alpha }{2}}+Z_{1-\beta }\right)}^{2}\left(s_{1}^{2}+s_{2}^{2}\right)}{{\left(\mu_{1}-\mu_{2}\right)}^{2}}\approx 20$$

The study involved 48 hands/patients with CTS. Using a simple randomization method and a random number table, participants were allocated into two groups: Ultrasound (24 hands/patients) and neural-gliding techniques (24 hands/patients). A checklist was used to obtain demographic information, weight, height, dominant hand, history of underlying disease, duration of CTS symptoms, severity of the disease (mild or moderate), distal motor and sensory latency of the median nerve before and after the intervention, pain intensity score before and after the intervention, severity of symptoms (feeling of pain and paresthesia), and functional status before and after the intervention.

Performing median nerve gliding exercises can help alleviate pain, improve grip strength, and enhance performance for individuals suffering from CTS. Long-term ischemia can make nerves sensitive and lead to damage due to a lack of proper oxygenation. Neuromobilization can effectively reduce ischemia in the canal area, improve oxygenation, and lower pain and sensory-motor symptoms in CTS sufferers. Additionally, this technique helps the nerves regain their normal range of motion, reducing nerve adhesion and ischemic pain by improving oxygenation in the nerve.

One group was treated with pulsed ultrasound at a frequency of 1 MHz, with a current intensity of 1 watt/cm^2^, applied three times a week for 10 sessions, each session lasting 5 minutes. The second group underwent neural-gliding interventions three times a week for 10 sessions. Both groups were taught standard treatment, including the use of a wrist splint. Distal motor and sensory latency of the median nerve was determined using electromyography (EMG) and nerve conduction studies (NCS) before the interventions.

Evaluation of pain intensity was conducted using the VAS criteria before interventions, and the BOSTON Questionnaire was completed to assess the intensity of symptoms (pain and paresthesia) and the functional status of the patient. The questionnaire consists of two parts: The first part contains 11 questions to evaluate the severity of symptoms, and the second part has 8 questions to assess functional status. The questionnaire is scored on a 5-point Likert Scale.

The severity of symptoms dimension (questions 1 - 11) and the functional status dimension (questions 12 - 19) were interpreted as follows:

- Severity of symptoms: Non-symptomatic (1 - 11), mild (12 - 22), moderate (23 - 33), severe (34 - 44), and very severe (45 - 55).

- Functional status: Normal (less than 8), mild (9 - 16), moderate (17 - 24), severe (25 - 32), and very severe (33 - 40).

Following the interventions, distal motor and sensory latency of the median nerve was re-evaluated using NCS, along with the VAS criteria and the BOSTON Questionnaire. All collected data were entered into IBM SPSS Statistics for Windows, version 22 (IBM Corp., Armonk, N.Y., USA). Descriptive statistical indices (central tendency and dispersion) were used to report descriptive data. The Shapiro-Wilk test was employed to check normality. Mann-Whitney and Wilcoxon tests, chi-square, or Fisher's exact tests were utilized for analysis. The significance level for this study was set at P < 0.05.

## 4. Results

In this randomized clinical trial, 48 patients with CTS participated and were divided into two groups using a simple randomization method. One group was treated with ultrasound (n = 24), and the other with neural gliding techniques (n = 24). The average age of the patients in the ultrasound group was 46.58 years, and in the neural gliding group, it was 47.66 years (P = 0.725). Most participants in both groups were women (91.6% versus 8.4%). The mean BMI was similar between groups (P = 0.618). There was no significant difference in occupation (P < 0.05), underlying diseases (P < 0.05), or disease severity based on electrodiagnostic findings (P = 0.060) at the baseline of the study, as shown in Table 1 and Table 2. 

**Table 1.** Comparison of Frequency Distribution of Demographic Information of Patients Among Ultrasound Group (N = 24) and Neural Gliding Group (N = 24) ^a^

Variables	Neural Gliding	Ultrasound	P-Value ^b^
**Sex**			> 0.999
Male	2 (8.3)	2 (8.3)	
Female	22 (91.7)	22 (91.7)	
**Occupation**			0.131
Housekeeper	14 (58.3)	7 (29.2)	
Employee	4 (16.7)	3 (12.5)	
Others	4 (16.7)	8 (33.3)	
Retired	2 (8.3)	6 (25.0)	
**Body Mass Index (BMI)**			0.579
Low weight	0 (0.0)	9 (37.5)	
Normal	12 (50.0)	6 (25.0)	
Overweight	6 (25.0)	9 (37.5)	
Obese	6 (25.0)	0 (0.0)	
**Underlying diseases**			0.819
Non	14 (58.3)	16 (66.7)	
Rheumatoid arthritis	2 (8.3)	3 (12.5)	
Diabetes mellitus	4 (16.7)	3 (12.5)	
Hypothyroidism	4 (16.7)	2 (8.3)	
**Dominant hand**			0.030
Right	20 (83.3)	12 (50.0)	
Left	4 (16.7)	12 (50.0)	
**Carpal tunnel syndrome severity**			0.060
Mild	4 (16.7)	11 (45.8)	
Moderate	20 (83.3)	13 (54.2)	

^a^ Values are expressed as No.(%).

^b^ Chi-square test.

**Table 2.** Comparison of Median (Q1 - Q3) Body Mass Index (BMI) and Symptom Duration of Patients Among Ultrasound Group (N = 24) and Neural Gliding Group (N = 24)

Variables	Neural Gliding	Ultrasound	P-Value
**Body Mass Index (BMI)**	25.5 (24.25 - 29.25)	27 (24.25 - 30.0)	0.618
** Symptoms duration**	12 (6.0 - 48.0)	36 (12.0 - 6.0)	0.048

The two groups were compared in terms of the average symptom severity score and function score measured via the BOSTON Questionnaire, sensory and motor median nerve latency, and pain score (VAS criteria) at the beginning of the study, indicating no significant difference between them (P > 0.05). Examination of the parameters at the end of the study compared to the baseline in the ultrasound group indicated a significant reduction in the average symptom severity score and function score measured via the BOSTON Questionnaire, showing improvement in symptom severity and nerve function (P < 0.001). The electrodiagnostic findings at the end of the study in this group revealed that the average sensory and motor median nerve latency was significantly reduced (P < 0.001). Additionally, the pain score (VAS criteria) significantly diminished post-intervention (P < 0.001).

The analysis of the parameters in the nerve gliding group at the end of the study compared to the beginning indicated a significant reduction in the average symptom severity score and function score measured via the BOSTON Questionnaire, along with the pain score based on VAS criteria (P < 0.001). However, there was no significant decline in the average sensory and motor median nerve latency in this group (P > 0.05). Comparison of the parameters between the two groups (nerve gliding and ultrasound) at the end of the study indicated no significant difference in the average symptom severity score and function score measured via the BOSTON Questionnaire (P > 0.05) and no significant difference in pain score based on VAS criteria (P > 0.05). However, the median nerve sensory and motor latency score in the ultrasound group was significantly lower than in the nerve gliding group (P < 0.050), as shown in Table 3. 

**Table 3.** Comparison of Median (Q1 - Q3) Symptoms Severity, Function Score, Sensory and Motor Median Nerve Latency, and Pain Score of Patients Among Ultrasound Group (N = 24) and Neural Gliding Group (N = 24)

Variables	Neural Gliding	Ultrasound	P-Value ^a^
**Symptoms severity score at before**	23.5 (12.5 - 35.75)	31.5 (23.5 - 39.0)	0.179
**Symptoms severity score after**	12.5 (11.0 - 16.0)	13.0 (11.0 - 16.0)	0.688
**P-value**	P < 0.001	P < 0.001	
**Function score before**	20.5 (12.5 - 27.0)	22 (13.5 - 25.0)	0.951
**Function score after**	10.0 (8.0 - 12.75)	11.0 (8.25 - 13.75)	0.588
**P-value**	< 0.001	< 0.001	
**Sensory median nerve latency before**	4.6 (4.22 - 5.3)	4.2 (3.9 - 4.77)	0.062
**Sensory median nerve latency after**	4.55 (4.2 - 5.27)	3.85 (3.6 - 4.27)	< 0.001
**P-value**	0.087	< 0.001	
**Motor median nerve latency before**	4.5 (4.22 - 5.37)	4.45 (4.1 - 4.77)	0.358
**Motor median nerve latency after**	4.45 (4.2 - 5.37)	4.0 (3.6 - 4.4)	0.004
**P-value**	0.467	0.001	
**Pain score before**	4.0 (2.0 - 6.75)	6.0 (2.0 - 6.75)	0.582
**Pain score after**	1.5 (1.0 - 3.0)	2.0 (0.25 - 4.0)	0.983
**P-value**	< 0.001	< 0.001	

^a^ Mann Whitney U test.

## 5. Discussion

As mentioned in the introduction, CTS is the most common entrapment neuropathy, causing significant disability in fine handwork. Consequently, numerous studies have been conducted on the treatment of CTS. However, there is still no consensus on the best conservative treatment for mild to moderate CTS, and further studies are required to determine their effectiveness. Among the conservative treatments are ultrasound and nerve gliding.

According to the results of the present study, the average sensory and motor median nerve latency in the ultrasound group was significantly reduced compared to the baseline of the study. However, this significant reduction was not observed in the nerve gliding group. In the study conducted by Ciechanowska (25), it was reported that the mean distal sensory and motor latency of the median nerve in patients receiving ultrasound therapy showed a significant decrease compared to the beginning of the study, which was consistent with our results. Additionally, the results observed in other studies conducted by Piravej and Boonhong (26) and Bakhtiari and Rashidy-Pour (6) were concordant with our findings in the ultrasound group. Another study performed by Atya and Mansour (24) showed that there was no significant improvement in electrodiagnostic findings in the nerve gliding group at the end of the study, which was consistent with our study. These results suggest that ultrasound therapy can be effective in improving electrodiagnostic indicators, and further studies are recommended. However, in the studies by Erol Oten (27) and Oztas et al. (28), no significant improvement in electrodiagnostic findings was observed in the ultrasound group at the end of the study, which was not consistent with our findings. The reason for this difference could be related to the type, intensity, and duration of ultrasound used.

At the end of the present study, in both groups compared to the baseline, the average pain score (VAS criteria) diminished significantly, with no significant difference between the ultrasound and nerve gliding groups. Ciechanowska (25) and Piravej and Boonhong (26) reported that the average pain score of the examined patients in the ultrasound group had a significant decline, which was consistent with our study. Additionally, in the study conducted by Wolny et al. (29), it was reported that at the end of the study, the mean pain score in the patients of the two groups (neural mobilization and electrophysical modality) had a significant reduction, which was in line with our findings. Furthermore, Alam et al. (30) showed that nerve gliding was more beneficial than ultrasound therapy in reducing pain intensity in CTS patients, which was not in accordance with our results. The difference between this study and our results could be due to the lower intensity of ultrasound used in their study. These results suggest that both ultrasound and nerve gliding can reduce pain intensity in patients with mild to moderate CTS.

At the end of the study, the average symptom severity and function scores measured via the BOSTON Questionnaire in both groups were significantly reduced compared to the beginning of the study, with no significant difference between the ultrasound and nerve gliding groups. Abdolrazaghi et al. (31) reported that the severity of disease symptoms in patients with CTS receiving nerve gliding interventions was significantly reduced, which was consistent with our study. Thanaya et al. (32) and Asadi et al. (33) demonstrated that the average symptom severity score (BOSTON Questionnaire) in patients receiving ultrasound had a significant decline, similar to our results. Additionally, similar results to our study were observed in the studies by Wolny et al. (29), Page et al. (34), Ansar et al. (35), and Bakhtiari and Rashidy-Pour (6). According to these findings, ultrasound and nerve gliding can improve nerve function and symptom severity in CTS patients. However, in the study conducted by Oten (27), there was no significant improvement in nerve function and symptom severity in the ultrasound group at the end of the study, which was not in line with our results. The small number of patients in their study may account for this difference.

Ultrasound therapy led to the improvement of symptoms and neurological function in the patients. The exact mechanism of the therapeutic effects of ultrasound in CTS treatment is unclear (33). Based on the findings of previous studies, ultrasound leads to tissue destruction and has anti-inflammatory effects (36). Different types of ultrasound treatment methods, including pulsed ultrasound (PUS) and continuous ultrasound (CUS), reduce the inflammation around the nerve, accelerate the healing of the damaged tissue, and contribute to the improvement of the patient's symptoms and nerve conduction by reducing the pressure inside the carpal tunnel (6).

Neural gliding techniques involve moving the nerve throughout the available range of motion (37). The efficacy of this method is not entirely clear, but there is evidence showing nerve gliding can be effective in reducing adhesions, improving oxygenation, and decreasing pain by allowing the nerve to move freely in patients suffering from CTS (22).

In conclusion, both ultrasound and nerve gliding techniques effectively reduce patients' symptoms and pain intensity in the short term. Nevertheless, ultrasound has the additional ability to improve electrodiagnostic indicators and appears more beneficial than nerve gliding. Considering the effects of both ultrasound and nerve gliding, these methods can be used alongside other conservative treatments such as splints, local steroid injections, and pharmacotherapy. Additionally, due to the effects of nerve gliding in improving patients' symptoms and pain intensity, it can be used as a simple and inexpensive method in combination with other treatments. A combination of ultrasound and nerve gliding may be more effective in treating CTS. However, further studies with a larger number of patients and extended follow-up periods are required to discover the long-term effects of these two modalities and their combination.

## Data Availability

The dataset presented in the study is available on request from the corresponding author during submission or after publication. The data are not publicly available because of ethics.

## Clinical Trial Registration Code:

IRCT20190618043934N23.
